# Cytokine and immunoglobulin production by PWM-stimulated peripheral and tumor-infiltrating lymphocytes of undifferentiated nasopharyngeal carcinoma (NPC) patients

**DOI:** 10.1186/1471-2407-4-68

**Published:** 2004-09-27

**Authors:** Lilia Fliss-Jaber, Radhia Houissa-Kastally, Kamel Bouzouita, Naceur Khediri, Ridha Khelifa

**Affiliations:** 1 Service des Laboratoires, Hôpital Habib Thameur, Tunis, Tunisia; 2 Laboratoire National de Contrôle des Médicaments, Tunis, Tunisia

## Abstract

**Background:**

Undifferentiated Nasopharyngeal Carcinoma (NPC) patients show a characteristic pattern of antibody responses to the Epstein-Barr virus (EBV) which is regularly associated with this tumor. However, no EBV-specific cytotoxic activity is detectable by the standard chromium-release assay at both peripheral and intratumoral levels. The mechanisms underlying this discrepancy between the humoral and cellular immune responses in NPC are still unknown, but might be related to an imbalance in immunoregulatory interleukin production. In this report, we investigated the ability of peripheral (PBL) and tumor- infiltrating (TIL) lymphocytes of undifferentiated NPC patients to produce in vitro three interleukins (IL-2, IL-6, IL-10) and three immunoglobulin isotypes (IgM, IgG, IgA).

**Methods:**

Lymphocytes from 17 patients and 17 controls were cultured in the presence of Pokeweed mitogen (PWM) for 12 days and their culture supernatants were tested for interleukins and immunoglobulins by specific enzyme-linked immunosorbent assays (ELISA). Data were analysed using Student's t-test and probability values below 5% were considered significant.

**Results:**

The data obtained indicated that TIL of NPC patients produced significantly more IL-2 (p = 0,0002), IL-10 (p = 0,020), IgM (p= 0,0003) and IgG (p < 0,0001) than their PBL. On the other hand, patients PBL produced significantly higher levels of IL-2 (p = 0,022), IL-10 (p = 0,016) and IgM (p = 0,004) than those of controls. No significant differences for IL-6 and IgA were observed.

**Conclusion:**

Taken together, our data reinforce the possibility of an imbalance in immunoregulatory interleukin production in NPC patients. An increased ability to produce cytokines such as IL-10 may underlie the discrepancy between humoral and cellular immune responses characteristic of NPC.

## Background

Undifferentiated nasopharyngeal carcinoma (NPC) is a malignant epithelial tumor characterized by a heavy infiltration of non malignant lymphocytes and most of these tumor infiltrating lymphocytes (TIL) have been shown to be T cells [1].

The Epstein-Barr virus (EBV) is causally associated with this malignancy since viral DNA is regularly present in the malignant epithelial cells but not in the neighbouring normal tissues. In addition, NPC patients show a specific pattern of humoral responses against EBV antigens [2].

Viral proteins known to be expressed in NPC tumor cells are the EBV-encoded nuclear antigen 1 (EBNA-1) and the latent membrane proteins LMP-1 in 35 to 65% of cases, and LMP-2 [3,4]. The latent membrane proteins have been shown to serve as targets for EBV-specific cytotoxic T lymphocytes (CTL) from normal seropositive individuals [5,6].

Recently, CD8 positive EBV-specific cytotoxic T cell clones were isolated from the peripheral blood and tumors of NPC patients [7]. The majority of the isolated CTL clones are directed towards the most immunogenic EBNA3 proteins which are not expressed in NPC tumor cells. No EBV-specific CTL activity is detectable by the standard chromium release assay in NPC patients [8-10] and the activity of any CTLs that would be present in such patients appears to be somehow suppressed. This lack of cytotoxic activity is in sharp contrast with the strong anti-EBV humoral immune response seen in patients [11,12]. The discrepancy between these two types of immune responses in NPC is still unexplained. It has been hypothesized that some viral gene products might have the capacity to influence cytokine production in such a way as to inhibit specific CTL activity [3,13]. Interestingly, the product of the EBV BCRF1 open reading frame has been found to display extensive homology with human interleukin 10 [IL-10 ; [14]]. Like its human counterpart, this viral product designated vIL-10, exerts immunosuppressive functions [15]. It is postulated that IL-10 production in malignant tumors may facilitate their escape from immune surveillance [16].

The expression of IL-10 in NPC has been controversial. While it has been reported that IL-10 is not expressed by NPC cells as detected by RNA in situ hybridisation [17], some reports using immunohistochemical and molecular techniques showed the expression of this cytokine by epithelial NPC tumor cells and TIL [18-20]. These authors suggested IL-10 as a possible evasion mechanism against the host antiviral system. Such a mechanism would explain the lack of detection of EBV specific cytotoxic activity in NPC patients at both peripheral and intratumoral levels [8-10,21]. Indeed, IL-10 is known to inhibit cell-mediated immune responses [22]. IL-10 is also known for upregulating the B cell response [23] and therefore, this putative mechanism is in accordance with the strong EBV-specific humoral immune response seen in NPC [11,12,24]. Other interleukins such as IL-2 and IL-6 may also appear to be involved in this discrepancy between humoral and cellular immune responses due to their central regulatory effects on T or B cells [25,26].

In this report, we investigated the ability of both peripheral blood lymphocytes (PBL) and TIL of undifferentiated NPC patients to express three interleukins (IL-2, IL-6, IL-10) and three immunoglobulin isotypes (IgM, IgG, IgA) following pokeweed mitogen (PWM) stimulation in vitro.

The data obtained indicated some significant differences between NPC patients and controls in interleukin and immunoglobulin production. However, further investigations are needed to establish the relevance of these differences to the discrepancy between humoral and cellular immune responses characteristic of NPC.

## Methods

### Patients and controls

17 untreated Tunisian NPC patients were included in this study. Informed consent was obtained from all patients before collection of blood and biopsy samples. These patients presented for treatment at the National Cancer Institute Salah Azaiez, Tunis or at the Farhat Hached hospital, Sousse, Tunisia. Their mean age was 47,5 years (range 25–70). All patients were diagnosed histopathologically as undifferentiated NPC.

17 healthy EBV carriers who presented to donate blood at the National Blood Transfusion Centre, Tunis, Tunisia, were included as controls in this study.

Sera of all patients and controls were titrated for antibodies directed against EBV antigens by indirect immunofluorescence [27].

### Lymphocyte preparations

Peripheral blood and biopsy samples were taken on the same day from each of the 17 NPC patients. Peripheral blood was collected from patients and healthy donors in heparin. Mononuclear cells were isolated by centrifugation over Ficoll-Hypaque (Pharmacia) according to Boyum [28]. Following three washes in RPMI-1640 medium (Gibco), cells were resuspended in RPMI-1640 medium supplemented with 10% heat-inactivated fetal calf serum (FCS ; Gibco) and 50 μg/ml Gentamicin (complete medium) at a concentration of 2 × 10^6 ^cells/ml.

For the preparation of tumor-infiltrating lymphocytes, biopsies were collected from NPC tumors under sterile conditions and immediately transferred to RPMI-1640 medium supplemented with 50 μg/ml Gentamicin. The biopsies were washed several times in this medium and then minced into small pieces in order to extract lymphocytes. The extracted cells were then washed by centrifugation in the same medium and then resuspended in complete medium at a final concentration of 2 × 10^6 ^cells/ml.

Samples from each lymphocyte preparation were taken for immunophenotyping by indirect membrane immunofluorescence using mouse monoclonal antibodies directed against CD3, CD4, CD8 or CD19 and FITC-conjugated goat anti-mouse F(ab')_2 _fragment. The labelled cells were counted under a fluorescence microscope.

### Stimulation by Pokeweed mitogen

PBL from healthy donors or NPC patients and TIL from the same patients suspended at 2 × 10^6 ^cells/ml in complete medium were distributed at 1 ml / well in 24-well Costar plates. One hundred microliters of a predetermined optimal dilution of PWM (Gibco), for treated wells, or complete medium alone, for untreated controls, were added in corresponding wells. The cells were then cultured for 12 days at 37°C, 5% CO_2 _and 98% relative humidity in a CO_2 _incubator. At the end of this incubation period, culture supernatants were harvested and used for cytokine and immunoglobulin determination as described below.

### Cytokine and Immunoglobulin determination

Culture supernatants of PBL and TIL stimulated by PWM under optimal conditions were used for both cytokine and immunoglobulin determination by enzyme-linked immunosorbent assays (ELISA).

IL-2, IL-6 and IL-10 concentrations were measured using commercial sandwich-type ELISA kits (Immunotech enzyme immunoassays, France) according to the procedures described by the manufacturer.

IgM, IgG and IgA concentrations were measured using sandwich-type ELISA assays prepared in our laboratory. Briefly, 96 well-microplates (Greiner, Germany) were coated for 2 hours at 37°C and overnight at 4°C with 150 μl/well of isotype-specific mouse monoclonal antibody (Sigma, France) appropriately diluted in 0,1 M carbonate buffer, pH 9.6. The microplates were washed four times with phosphate-buffered saline pH 7.4 containing 0,1 % Tween 20 (PBS-Tween), then blocked with 300 μl/well of PBS containing 1% FCS for 30 minutes at 37°C. For the assay, 100 μl /well of appropriately diluted culture supernatants in PBS-Tween containing 10% FCS were incubated in coated microplates for 2 hours at 37°C. The microplates were then washed four times with PBS-Tween and 100 μl/well of the appropriate alkaline phosphatase-labeled conjugate (Goat anti-human IgM, IgG or IgA, Sigma-France) were added at a 1:20000 dilution in PBS containing 1% FCS. After a two-hour incubation at 37°C, the microplates were washed and a p-nitrophenylphosphate substrate solution was added. The microplates were incubated for 1 hour at 37°C and the reaction was stopped with 50 μl/well of 1 N NaOH. Immunoglobulin standards were generated using purified human immunoglobulins of each isotype (Sigma, France). The microplates were read at 405 nm on a microplate reader (LP-400, Diagnostic Pasteur). Data were represented as the mean immunoglobulin concentration of triplicate cultures. Immunoglobulin values were obtained by interpolation from standard curves.

### Statistical analysis

Data were analysed using "Student's t-test". Probability values below 5% were considered significant.

## Results

### EBV serology

All patients showed a typical serological profile characteristic of NPC as determined by indirect immunofluorescence. Levels of IgG antibodies to VCA were considerably higher than those in healthy donors. Their titers varied from 640 to 2560 (mean titer = 1065), whereas in healthy donors such titers fluctuated between 40 and 160 (mean titer = 70). IgG antibodies to EA were present in NPC patients only, with titers ranging from 20 to 320 (mean titer = 50).

Anti-VCA IgA antibodies were detected in all patients (range : 20–320 ; mean = 61) and anti-EA IgA antibodies were detected in only 4 patients (range : 10–80 ; mean = 27). None of the controls showed IgA antibodies against these antigens.

### Immunophenotyping of lymphocyte preparations

Immunophenotypic analysis of the lymphocyte preparations obtained from patients and controls indicated that CD3+ lymphocytes constituted the major subpopulation with a mean frequency of (60 ± 8)% in PBL of both patients and controls and (55 ± 9)% in TIL. CD4+ lymphocytes represented (35 ± 5)% in PBL of both patients and controls and (20 ± 3)% in TIL. CD8+ cells showed mean values of (40 ± 6)% in PBL of patients, (36 ± 4)% in TIL, and (28 ± 5)% in PBL of controls.

On the other hand, CD19+ B lymphocytes represented in average (25 ± 5)% in PBL of patients, (23 ± 6)% in TIL, and (22 ± 3)% in PBL of controls.

### Cytokine production

**Figure 1:**
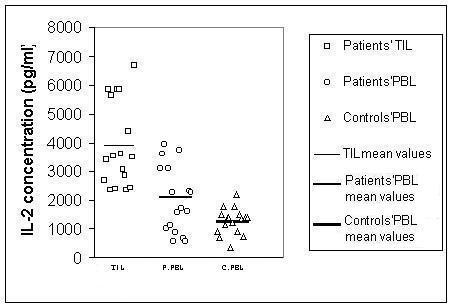
IL-2 production. PBL and TIL from 17 NPC patients and PBL from 17 controls were cultured at a concentration of 2 × 10^6 ^cells/ml in the presence of PWM at 1:200 dilution for 12 days. Culture supernatants were collected and assayed for IL-2.

**Figure 2:**
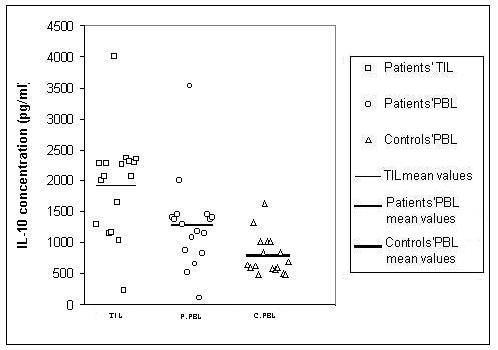
IL-10 production. PBL and TIL from 17 NPC patients and PBL from 17 controls were cultured at a concentration of 2 × 10^6 ^cells/ml in the presence of PWM at 1:200 dilution for 12 days. Culture supernatants were collected and assayed for IL-10.

**Figure 3:**
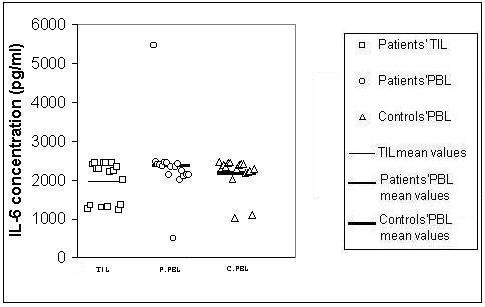
IL-6 production. PBL and TIL from 17 NPC patients and PBL from 17 controls were cultured at a concentration of 2 × 10^6 ^cells/ml in the presence of PWM at 1:200 dilution for 12 days. Culture supernatants were collected and assayed for IL-6.

As illustrated in Figures 1 and 2, the PBL of NPC patients produced significantly more IL-2 (2105 ± 1152 pg/ml) and IL-10 (1280 ± 727 pg/ml) than the PBL of healthy donors (1286 ± 452 pg/ml, p = 0,022 for IL-2 and 793 ± 325 pg/ml, p = 0,016 for IL-10). Comparable amounts of IL-6 were found for both groups (2375 ± 919 pg/ml for patients and 2177 ± 435 pg/ml for controls, p = 0,429 ; Figure 3). On the other hand, TIL of patients showed a significantly higher IL-2 (3913 ± 1484 pg/ml, p = 0,0002) and IL-10 (1926 ± 817 pg/ml, p = 0,02) production than their PBL. The observed differences in mean IL-6 production between patients'PBL (2375 ± 919 pg/ml) and TIL (1962 ± 515 pg/ml) did not reach statistical significance (p = 0,116).

Unstimulated lymphocytes cultured in the absence of PWM did not show any detectable cytokine production (data not shown).

### Immunoglobulin production

**Figure 4:**
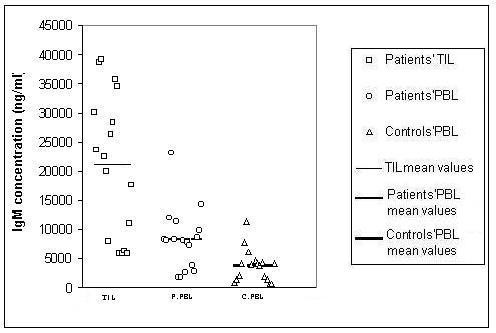
IgM production. PBL and TIL from 17 NPC patients and PBL from 17 controls were cultured at a concentration of 2 × 10^6 ^cells/ml in the presence of PWM at 1:200 dilution for 12 days. Culture supernatants were collected and assayed for IgM.

The results of immunoglobulin determination showed that the PBL of NPC patients produced significantly higher levels of IgM (8262 ± 5315 ng/ml) than the PBL of controls (3753 ± 2801 ng/ml, p = 0,004 ; Figure 4).

**Figure 5:**
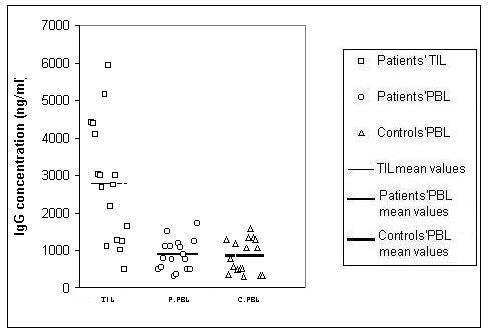
IgG production. PBL and TIL from 17 NPC patients and PBL from 17 controls were cultured at a concentration of 2 × 10^6 ^cells/ml in the presence of PWM at 1:200 dilution for 12 days. Culture supernatants were collected and assayed for IgG.

**Figure 6:**
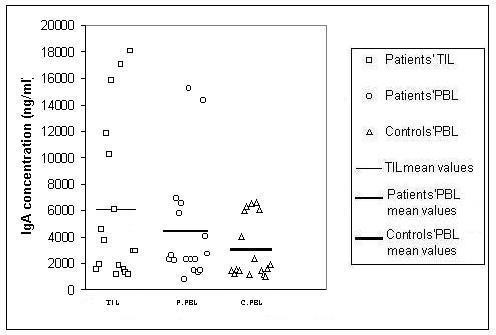
IgA production. PBL and TIL from 17 NPC patients and PBL from 17 controls were cultured at a concentration of 2 × 10^6 ^cells/ml in the presence of PWM at 1:200 dilution for 12 days. Culture supernatants were collected and assayed for IgA.

Both groups produced similar amounts of IgG (874 ± 408 ng/ml for patients and 847 ± 442 ng/ml for controls, p = 0,85 ; Figure 5) and IgA (4380 ± 4316 ng/ml for patients and 3067 ± 2267 ng/ml for controls, p = 0,27 ; Figure 6). On the other hand, TIL of these patients produced significantly higher levels of IgM (21162 ± 12276 ng/ml, p = 0,0003) and IgG (2789 ± 1583 ng/ml, p < 0,0001) than their PBL. No significant differences in IgA production were found between PBL (4380 ± 4316 ng/ml) and TIL (6112 ± 6046 ng/ml, p = 0,34).

Unstimulated lymphocytes cultured in the absence of PWM did not show any detectable immunoglobulin production (data not shown).

## Discussion

Previous reports indicate the absence of EBV-specific cytotoxic T lymphocytes detectable by the standard chromium release assay in both the peripheral and intratumoral compartments [8-10,21] in undifferentiated nasopharyngeal carcinoma. However, NPC patients show a strong EBV-specific humoral immune response, and elevated titers of anti-EBV antibodies directed against viral antigens are observed in their sera [11,12,24]. The mechanisms underlying this discrepancy between humoral and cellular immune responses in NPC patients are still unknown. It was postulated that interleukin 10 production in malignant tumors would facilitate their escape from immune surveillance [16].

The expression of IL-10 in NPC has been controversial. While some authors have reported that NPC tumor cells do not express IL-10 [17], others observed IL-10 expression in epithelial NPC tumor cells and tumor infiltrating lymphocytes [18,19]. They suggested such IL-10 expression as a possible mechanism for NPC tumors and EBV to escape local cellular immune attack. Indeed, IL-10 is a pleiotropic factor known for its suppressive effects on cell-mediated immune responses [22]. In sharp contrast to these inhibitory effects, IL-10 also has a potent stimulatory effect on the humoral immune response, inducing B lymphocyte differentiation and immunoglobulin secretion [23]. Therefore, IL-10 hyperproduction alone or in association with changes in other cytokines might lead to an imbalance between humoral and cellular immune responses similar to that seen in NPC.

The expression of cytokines such as IL-2 [19], IL-6 [17] and IL-10 [18,19] in NPC has been studied mainly on tumor biopsies using immunohistochemical and molecular techniques. To the best of our knowledge, only one study on the ability of NPC patients'lymphocytes to produce cytokines in culture has been reported and it was limited to IL-2 [9].

In this report, we investigated the ability of both peripheral blood and tumor infiltrating lymphocytes of 17 undifferentiated NPC patients to produce cytokines following mitogenic stimulation in culture. Since immunoglobulin isotypes are determined by cytokine patterns, we also looked for possible correlations between measured cytokine levels and immunoglobulin isotypes produced. PWM was chosen to stimulate the lymphocyte cultures because it is known to activate both T and B lymphocytes in humans [29,30]. In addition, the ability of PWM to stimulate cytokine production by Tcells is similar to that of PHA [31]. This allowed us to study the responses of both T and B cells simultaneously in each lymphocyte culture.

The data obtained indicate that the highest levels of IL-2 were produced by TIL cultures followed by patients'PBL. This is in line with a report by Lakhdar et al [9] showing a higher IL-2 production by PHA-stimulated PBL of NPC patients than by controls. Such high IL-2 levels are expected to favor a strong cytotoxic response since IL-2 is needed for CTL stimulation and proliferation [32] and they are not consistent with the known lack of detectable CTL activity.

IL-10, a representative of the Th2 pattern of cytokines, is generally considered immunosuppressive. It inhibits IL-6 secretion by activated macrophages but not by Th2 lymphocytes [33]. In the present work, IL-10 was overproduced by patients'lymphocyte cultures whereas their IL-6 levels were similar to controls, showing no signs of inhibition by IL-10. This points to Th2 lymphocytes rather than activated macrophages as the main source of IL-6 in this system. The increased ability of patients lymphocytes to produce IL-10 is compatible with the lack of CTL activity.

Cultures of tumor infiltrating lymphocytes secreted significantly higher amounts of IgM and IgG than the PBL of either patients or controls in good agreement with the increased levels of IL-2 and IL-10, since these cytokines are involved in B cell activation and enhance immunoglobulin synthesis [23,25]. No significant correlations between cytokine production and IgA secretion were found, in line with the preferential enhancing effect of IL-2 and IL-10 on IgM and IgG synthesis [23].

In an attempt to see whether the observed differences in cytokine and immunoglobulin production between patients and controls or between PBL and TIL of patients could be due to differences in the composition of individual lymphocyte preparations, we performed an immunophenotypic analysis of each lymphocyte preparation. As shown in the results, the only significant changes in the proportions of lymphocyte subsets between patients and controls were observed in the CD8 subpopulation which showed an increase in patients PBL and TIL. On the other hand, the CD4 subset showed a significant decrease in TIL. Such differences in lymphocyte subpopulations are not expected to produce the changes in cytokine and immunoglobulin production observed here, since CD4 lymphocytes are well known for being the main source of IL-2 and IL-10, and for their ability to stimulate immunoglobulin synthesis [34].

Recently, CD4+ lymphocyte populations have been shown to be more heterogeneous and complex than previously thought [35] and new subsets of T regulatory cells (Tr) with immunosuppressive activities have been identified. Tr cells have been suggested to play a key role in the evasion from immune-mediated clearance of microorganisms and tumors [36]. It has also been reported that IL-10-producing Tr1 cells dominate the immune response to LMP1 in EBV seropositive subjects [36]. Since LMP1 is expressed in NPC tumors, it would be tempting to speculate that the increase in IL-10 production by TIL would correspond to an increase in Tr1 cells in the corresponding NPC tumors. In this respect, Hodgkin lymphoma infiltrating lymphocytes have been shown to contain large populations of both Tr1 and CD4+CD25+ regulatory T cells [37], and it would be interesting to see whether a similar situation occurs in NPC.

## Conclusion

In conclusion, our data point to the possibility of an imbalance in immunoregulatory interleukin production in NPC patients. Their lymphocytes, especially those infiltrating the tumors, showed in particular a high propensity to produce IL-10 following mitogenic stimulation in vitro. Overproduction of this pleiotropic cytokine may lead to a discrepancy between humoral and cellular immune responses similar to that seen in NPC. The relevance of the present observations to the in vivo situation is not yet established and warrants further investigations.

## Competing interests

The authors declare that they have no competing interests.

## Authors' contributions

LFJ designed and carried out the experimental work, performed the statistical analysis and drafted the manuscript. NK maintained the cell cultures and provided technical help. RHK and KB participated in the design and coordination of the study. RK directed the research and finalized the manuscript.

All authors read and approved the final manuscript.

## Pre-publication history

The pre-publication history for this paper can be accessed here:



## References

[B1] Shanmugaratnam K, Chan SH, de-The G, Goh JE, Khor TH, Simons MJ (1979). Histopathology of nasopharyngeal carcinoma : correlations with epidemiology, survival rates and other biological characteristics. Cancer.

[B2] Zur Hausen H, Schulte-Holthausen H, Klein G (1970). EB-virus DNA in biopsies of Burkitt's tumors and anaplastic carcinomas of nasopharynx. Nature.

[B3] Brooks L, Yao QY, Rickinson AB, Young LS (1992). Epstein-Barr latent gene transcription in nasopharyngeal carcinoma cells: coexpression of EBNA1, LMP1 and LMP2 transcripts. J Virol.

[B4] Busson P, Mc Coy R, Sadler R, Gilligan K, Tursz T, Raab-Traub N (1992). Consistent transcription of the Epstein-Barr LMP2 gene in nasopharyngeal carcinoma. J Virol.

[B5] Lee SP, Tierney RJ, Thomas WA, Brooks JM, Rickinson AB (1997). Conserved CTL epitopes within EBV latent membrane protein 2. The Journal of Immunology.

[B6] Khanna R, Burrows SR, Nicholls J, Poulsen LM (1998). Identification of cytotoxic T cell epitopes within Epstein-Barr virus (EBV) oncogene latent membrane protein 1 (LMP1) : evidence for HLA A2 supertype-restricted immune recognition of EBV-infected cells by LMP1-specific cytotoxic T lymphocytes. Eur J Immunol.

[B7] Lee SP, Chan AT, Cheung ST, Thomas WA, Croom Carter D, Dawson CW, Tsai CH, Leung SF, Johnson PJ, Huang DP (2000). CTL control of EBV in nasopharyngeal carcinoma (NPC) : EBV-specific CTL responses in the blood and tumors of NPC patients and the antigen-processing function of the tumor cells. J Immunol.

[B8] Chan SH, Chew TS, Kunaratnam N (1979). Cell-mediated immunity to Epstein-Barr virus in nasopharyngeal carcinoma. The Lancet.

[B9] Lakhdar M, Ellouz R, Kammoun H, Ben H'tira S, Khediri N, Kastally R, Fridman WH (1987). Presence of in vivo activated T-cells expressing HLA-DR molecules and IL-2 receptors in peripheral blood of patients with nasopharyngeal carcinoma. Int J Cancer.

[B10] Lakhdar M, Thameur H, Maalej H, Ben Ayed F, Ladgham A (1993). Emergence of non major histocopatibility complex restricted lytic CD8+ cells in the blood of naspharyngeal carcinoma. Cancer Immunol Immunother.

[B11] Henle G, Henle W, Klein G (1971). Demonstration of two distinct components in the early antigen complex of Epstein-Barr virus-infected cells. Int J Cancer.

[B12] Henle W, Ho JHS, Henle G, Chan JC, Kwan HC (1977). Nasopharyngeal carcinoma : significance of changes in Epstein-Barr virus-related antibodies patterns following therapy. Int J Cancer.

[B13] Cochet C, Martel-Renoir D, Grunewald V, Bosq J, Cochet G, Schwaab G, Bernaudin JF, Joab I (1993). Expression of the Epstein-Barr virus immediate early gene, BZLF1, in nasopharyngeal carcinoma tumor cells. Virol.

[B14] Moore KW, Vieira P, Fiorentino DF, Trounstine ML, Khan TA, Mosmann TR (1990). Homology of cytokine synthesis inhibitory factor (IL-10) to the Epstein-Barr virus gene BCRF1. Science.

[B15] Hsu DH, de Waal Malefyt R, Fiorentino DF (1990). Expression of interleukin-10 activity by Epstein-Barr virus protein BCRF1. Science.

[B16] Salazar-Onfray F (1999). Interleukin-10 : a cytokine used by tumors to escape immunosurveillance. Med Oncol.

[B17] Beck A, Pazolt D, Grabenbauer GG, Nicholls JM, Herbst H, Young LS, Niedobitek G (2001). Expression of cytokine and chemokine genes in Epstein-Barr virus-associated nasopharyngeal carcinoma : comparison with Hodgkin's disease. J Pathol.

[B18] Yao M, Ohshima K, Suzumiya J, Kume T, Shiroshita T, Kikuchi M (1997). Interleukin-10 expression and cytotoxic-T-cell response in Epstein-Barr-virus-associated nasopharyngeal carcinoma. Int J Cancer.

[B19] Huang YT, Sheen TS, Chen CL, Lu J, Chang Y, Chen JY, Tsai CH (1999). Profile of cytokine expression in nasopharyngeal carcinoma. Cancer Res.

[B20] Fujieda S, Lee K, Sunaga H (1999). Staining of interleukin-10 predicts clinical outcome in patients with nasopharyngeal carcinoma. Cancer.

[B21] Tsukuda M, Sawaki S, Yanoma S (1993). Suppressed cellular immunity in patients with nasopharyngeal carcinoma. J Cancer Res Clin Oncol.

[B22] Moore KW, O'Garra A, de Waal Malefyt R, Vieira P, Mosmann TR (1993). Interleukin-10. Annu Rev Immunol.

[B23] Rousset F, Garcia E, Defrance T, Peronne C, Vezzio M, Hsu DH, Kastelin R, Moore KW, Banchereau J (1992). Interleukin-10 is a potent growth and differentiation factor for activated human B lymphocytes. Proc Natl Acad Sci USA.

[B24] Xu J, Ahmed A, D'addario M, Knafo L, Jones JF, Prazard U, Dolcetti R, Vaccher E, Menezes J (2000). Analysis and significance of anti-latent membrane protein-1 antibodies in the sera of patients with EBV-associated disease. J Immunol.

[B25] Morgan DA, Ruscetti FW, Gallo R (1976). Selective in vitro growth of T lymphocytes from normal human bone marrows. Science.

[B26] Muraguchi A, Hirano T, Tang B (1988). The essential role of B cell stimulating factor (BSF-2/IL-6) for the terminal differentiation of B-cells. J Exp Med.

[B27] Henle W, Henle G, Azjac BA, Pearson G, Waubke R, Scriba M (1970). Differential reactivity of human serums with early antigens induced by Epstein-Barr virus. Science.

[B28] Boyum M (1968). Isolation of mononuclear cells and granulocytes from human blood. Scand J Clin Lab Invest.

[B29] Ichiki Y, Takahashi W, Watanabe T (1992). The effect of cytokines and mitogens on the induction of C epsilon germline transcripts in a human Burkitt lymphoma B cell line. Int Immunol.

[B30] Hartnett BJ, Somberg RL, Krakowka S, Ochs HD, HogenEsch H, Moore PF, Wernberg KI, Felsburg PJ (2000). B-cell function in canine X-linked severe combined immunodeficiency. Vet Immunol Immunopathol.

[B31] Katial RK, Sachanandani D, Pinney C, Lieberman MM (1998). Cytokine production in cell culture by peripheral blood mononuclear cells from immunocompetent hosts. Clin Diagn Lab Immunol.

[B32] Suzuki R, Handa K, Itoh K, Kumagai K (1983). Natural killer (NK) cells as a responder to interleukin 2 (IL-2). J Immunol.

[B33] Ralph P, Nakoinz I, Sampson-Johannes A, Fong S, Lowe D, Min HY, Lin L (1992). IL-10, T lymphocyte inhibitor of human blood cell production of IL-1 and tumor necrosis factor. J Immunol.

[B34] Mosmann TR, Cherwinski H, Bond MW, Giedlin MA, Coffman RL (1986). Two types of murine helper T cell clone. I. Definition according to profiles of lymphokines activities and secreted proteins. J Immunol.

[B35] Roncarolo M, Levings MK (2000). The role of different subsets of T regulatory cells in controlling autoimmunity. Curr Opin Immunol.

[B36] Marshall NA, Vickers MA, Barker RN (2003). Regulatory T cells secreting IL-10 dominate the immune response to EBV latent membrane protein 1. J Immunol.

[B37] Marshall NA, Christie LE, Munro LR, Culligan DJ, Johnston PW, Barker RN, Vickers MA (2004). Immunosuppressive regulatory T cells are abundant in the reactive lymphocytes of Hodgkin lymphoma. Blood.

